# Discovery of spectosis supports the key role of caspase-8 in determining the type of cell death in mature erythrocytes and erythrocyte death-driven immunogenicity

**DOI:** 10.1038/s41420-026-02989-0

**Published:** 2026-02-21

**Authors:** Anton Tkachenko

**Affiliations:** 1 Department of Cryobiochemistry, Institute for Problems of Cryobiology and Cryomedicine of the National Academy of Sciences of Ukraine, Kharkiv, Ukraine; 2 BIOCEV, First Faculty of Medicine, Charles University, Vestec, Czech Republic

**Keywords:** Apoptosis, Necroptosis

## Abstract

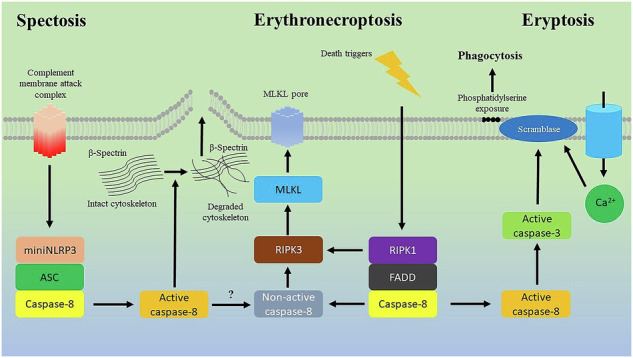

In 2018, the Nomenclature Committee on Cell Death (NCCD) summarized recent advances in the field of cell death and defined over a dozen distinct regulated cell death (RCD) modalities occurring in nucleated cells [1]. These RCD subroutines are known to be linked with a wide range of apoptotic (non-lytic) and necrotic (lytic) features on extreme ends of the morphological spectrum. Importantly, plasma membrane integrity is one of the key determinants of the immunostimulatory signals emitted by dying cells, since cell lysis enables the release of intracellular cytokines and damage-associated molecular patterns (DAMPs), thereby igniting local inflammation. Indeed, a wide spectrum of RCDs in nucleated cells can be associated with the varying kinetics of cytokine and DAMP release, determining the complexity of immunogenic cell death [2]. In contrast to nucleated cells, the landscape of cell death processes in mature erythrocytes (bereft of organelles that act as multifaceted regulators of cell death, e.g., mitochondria or lysosomes) has been unraveled to a lesser extent. In 2025, the Consortium for Erythrocyte Cell Death Research (CECDR) published a review article epitomizing RCD pathways of mature red blood cells (RBCs) and clearly demonstrating that erythrocytes succumbing to stress could undergo both non-lytic (eryptosis) and lytic cell death (erythronecroptosis) [3]. Eryptosis is a form of erythrocyte cell death mechanistically dependent on Ca^2+^ overload with subsequent scramblase-dependent phosphatidylserine (PS) externalization [3]. PS externalization ensures rapid erythrophagocytosis of eryptotic cells by macrophages disposing of the damaged RBCs. There is some evidence that eryptosis is an immunologically silent event (like apoptosis of nucleated cells) and PS exposed at the surface of eryptotic RBCs functions as an immunosuppressive signal that inhibits pro-inflammatory activity of macrophages following engulfment of PS-expressing eryptotic cells [4]. Erythronecroptosis or necroptosis of erythrocytes was firstly described in 2014 by LaRocca et al. as a lytic RIPK1/RIPK3/MLKL signaling-dependent form of RBC death triggered by human-specific bacterial pore-forming toxins [5]. Currently, the role of erythronecroptosis in health and disease, as well as its immunogenic characteristics, remains elusive. However, DAMPs released from dying nucleated necroptotic cells are well-known drivers of inflammation [6]. Thus, cell death of erythrocytes has important implications for the immune response.

It is becoming increasingly clear that caspase-8 is one of the central players in erythrocyte cell death signaling pathways. Notably, in contrast to apoptosis, eryptosis can be executed without activation of caspases. However, extrinsic Fas-mediated eryptosis is linked to the recruitment of caspase-8 and caspase-3 [3]. At the same time, caspase-8 inhibition is a prerequisite condition for erythronecroptosis [5]. Thus, the caspase-8 status has been postulated to determine a form of RBC death, thereby conceptually directing its immunogenicity [7]. Moreover, it has been hypothesized that the retention of caspase-3 and caspase-8, the only representatives of caspase family proteases in mature erythrocytes, during erythropoiesis is an evolutionary mechanism to ensure the crosstalk between eryptotic and necroptotic pathways to regulate RBC death-driven immune responses [8].

In 2025, Chen et al. reported that erythrocytes could undergo a third form of RCD termed “spectosis” [9]. This lytic RCD was triggered by the complement membrane attack complex (MAC) with subsequent formation of the NLRP3/ASC/caspase-8 complex and downstream degradation of β-spectrin. NLRP3 was represented by its truncated version (miniNLRP3). Notably, spectosis was linked to the release of heme, a pro-inflammatory erythrocyte-derived DAMP molecule, an event mediated by caspase-8-dependent cleavage of β-spectrin. Importantly, recruitment of NLRP3/ASC to spectosis indicates its certain resemblance to pyroptosis, a strongly pro-inflammatory cell death of nucleated cells linked to the release of IL-1β and IL-18 through gasdermin-forming pores. However, in nucleated cells, caspase-8 acts upstream of the NLRP3 inflammasome, acting as a positive modulator of the NLRP3 inflammasome signaling, while it acts as an executioner caspase in spectosis, directly responsible for β-spectrin degradation downstream of NLRP3 activation [9]. Intriguingly, the miniNLRP3/ASC/caspase-8 complex formed in spectosis resembles a PANoptosome complex composed of NLRP3, ASC, caspase-1, caspase-8, and RIPK3 reported to drive PANoptosis in macrophages [10]. PANoptosis, as a complex form of pro-inflammatory RCD orchestrated by PANoptosomes, shares features of apoptosis, pyroptosis, and necroptosis [11]. Strikingly, the NLRP3/ASC/caspase-8/RIPK3 PANoptosome formation in macrophages can trigger PANoptosis in response to canonical NLRP3 triggers even without recruitment of caspase-1 and gasdermin D (GSDMD) [10] whose expression in mature erythrocytes is not reported. This increases similarity between spectosis in erythrocytes and PANoptosis in nucleated cells, which has been recently emphasized by Tweedell and Kanneganti [12]. Nevertheless, caspase-8 is involved in all three currently described RCDs of mature erythrocytes (eryptosis, erythronecroptosis, and spectosis) and disclosure of spectosis supports the role of caspase-8 in inflammatory signaling emitted by dying RBCs. This suggests that caspase-8 can be a key decision maker for eryptosis, erythronecroptosis, and spectosis in erythrocytes, like it is observed for apoptosis, necroptosis, pyroptosis, and PANoptosis in nucleated cells. However, a deeper understanding of caspase-8 function in mediating this crosstalk in mature erythrocytes is required.

Taken together, discovery of spectosis has broadened our understanding of the molecular basis for erythrocyte cell death signaling, delineates the same decisive regulatory role of caspase-8 in a plethora of tightly interconnected regulatory cascades involved in RCD execution, and expands the scope of mechanisms implicated in erythrocyte-driven regulation of the human immune response. Furthermore, the study by Chen et al. [9] provides insights into the intrinsic complexity of erythrocyte cell biology and emphasizes once again that investigation of erythrocyte RCDs is a potentially rich area of research, which might shed light on novel mechanisms of immune regulation. Indeed, further studies of the immunogenic consequences of RBC death pathways might open novel therapeutic avenues for a wide range of diseases. The NCCD considers erythrocytes as entities existing in a debatable state between life and death [1]. However, if they can undergo three distinct RCD modalities, adjusting their cell fate presumably via the caspase-8 status, RBCs should be treated as living cells. Thus, their demise is not a mechanistic disintegration but rather a tightly regulated biological event with far-reaching consequences.

## Data Availability

Data are available from the corresponding author upon reasonable request.
